# Dynamic super-resolution structured illumination imaging in the living brain

**DOI:** 10.1073/pnas.1819965116

**Published:** 2019-04-26

**Authors:** Raphaël Turcotte, Yajie Liang, Masashi Tanimoto, Qinrong Zhang, Ziwei Li, Minoru Koyama, Eric Betzig, Na Ji

**Affiliations:** ^a^Howard Hughes Medical Institute, Janelia Research Campus, Ashburn, VA 20147;; ^b^Department of Physics, University of California, Berkeley, CA 94720;; ^c^Department of Molecular & Cell Biology, University of California, Berkeley, CA 94720;; ^d^Helen Wills Neuroscience Institute, University of California, Berkeley, CA 94720;; ^e^Molecular Biophysics and Integrated Bioimaging Division, Lawrence Berkeley National Laboratory, Berkeley, CA 94720

**Keywords:** super-resolution, adaptive optics, brain imaging, in vivo, synapses

## Abstract

Cells in the brain act as components of extended networks. Therefore, to understand neurobiological processes in a physiological context, it is essential to study them in vivo. Super-resolution microscopy has spatial resolution beyond the diffraction limit, thus promising to provide structural and functional insights that are not accessible with conventional microscopy. However, to apply it to in vivo brain imaging, we must address the challenges of 3D imaging in an optically heterogeneous tissue that is constantly in motion. We optimized image acquisition and reconstruction to combat sample motion and applied adaptive optics to correcting sample-induced optical aberrations in super-resolution structured illumination microscopy (SIM) in vivo. We imaged the brains of live zebrafish larvae and mice and observed the dynamics of dendrites and dendritic spines at nanoscale resolution.

## Significance

The brain is composed of cells that continually communicate with one another via electric and chemical signals. To understand nerve cells in a physiological context, we must study them in vivo, for which optical microscopy is an essential tool. In particular, much of this communication takes place at the nanoscale level and requires in vivo super-resolution microscopy. We applied adaptive optics to correcting sample-induced optical aberrations and optimized image acquisition and reconstruction to combat sample motion, which allowed us to adapt super-resolution structured illumination microscopy to in vivo imaging in the brains of zebrafish larvae and mice. With these optimizations, we were able to image dynamic processes at dendrites and synapses in the mouse brain at nanoscale resolution in vivo.

In the brain, individual cells act as integral components of extended networks that are responsive to external inputs, modulated by internal states, and regulated by development and learning (1). As a result, to understand most neurobiological processes, we need to study cells in the intact brain. With subcellular spatial resolution, optical microscopy has long served as an essential tool for the investigation of brain structure and function in vivo. In recent years, super-resolution (SR) optical microscopy methods that provide even finer spatial detail have also been applied to neuroscience (2–7) and have revealed new structural insights in cultured cells or tissue samples. However, they have rarely been applied in vivo [but see the work by Pfeiffer et al. (8)] or at a temporal resolution that is sufficient to capture activity events in the brain.

Structured illumination microscopy (SIM) is a SR technique wherein multiple widefield images are acquired with grating illumination patterns of differing orientations and phases that are processed together to reconstruct a single SR image frame. SIM provides a good compromise for live cell SR imaging with respect to resolution, speed, labeling requirements, and photon budget. In its linear form, SIM achieves up to a twofold gain in spatial resolution at subsecond temporal resolution on samples labeled with conventional fluorescent dyes under physiological light intensities (9). Sufficient to resolve a plethora of subcellular processes, SIM has been implemented at high frame rates to monitor various dynamic events in vitro (10–13) and is thus a prime candidate for SR imaging of the brain in vivo.

SR imaging in an intact live brain poses several unique challenges due to its complex 3D fluorescence distribution, optical heterogeneity, and continual motion. We investigated how these factors impact SIM image formation and developed experimental strategies to mitigate their effects. Specifically, we combined adaptive optics (AO) with SIM to eliminate sample-induced aberrations, used subdiffractive objects to accurately evaluate illumination parameters, and used phase up-sampling and image registration to alleviate the effect of brain motion. The resulting optimization of data acquisition and analysis allowed us to apply SIM to in vivo imaging in the brain of the mouse and larval zebrafish and demonstrate high-speed (9.3 frames per second) robust imaging of synapses with SR (190 ± 11 nm).

## Results

### Experimental Setup of an in Vivo SR Structured Illumination Microscope.

The optical system for in vivo SIM imaging of the brain consisted of two modules: one for SIM itself and one for AO to compensate for specimen-induced aberrations (*SI Appendix*, Figs. S1*A* and S2). Each SIM frame was constructed from nine widefield fluorescent images (the “raw data series”) acquired with harmonic illumination excitation patterns of three different orientations and three phases at each orientation (*SI Appendix*, Fig. S1*B*). The SIM module generated these patterns at the sample by reflecting the light from a visible laser off a spatial light modulator located conjugate to the sample and programmed to present a corresponding harmonic pattern. The light then passed through the AO module, where it reflected off a pupil-conjugate deformable mirror before entering the objective lens pupil to excite patterned fluorescence over a wide field within the sample (*SI Appendix*, Fig. S1*C*). This fluorescence was then collected by the objective, corrected for aberrations on reflection off the deformable mirror, and directed to a sample-conjugate camera in the SIM module. To determine the corrective pattern to apply on the deformable mirror, the SIM unit was bypassed, and a pulsed laser was used for the generation of a nonlinear “guide star” via multiphoton excitation of the sample fluorescence with wavefront that was measured by a Shack–Hartmann sensor in the AO module.

### Accurate Determination of the Illumination Parameters Using Subdiffraction-Limited Beads.

SIM down-modulates sample spatial frequencies up to twice beyond the diffraction limit into the conventional diffraction-limited passband of the microscope via the mixing (i.e., difference frequency generation) between the sample and illumination spatial frequencies. The reconstruction of SIM images from the raw data series, therefore, requires precise knowledge of the harmonic illumination parameters: modulation depth (a), illumination vector describing the grating spatial frequency and orientation ($\vec{p}$), and phase ($\phi_{n}$) (*SI Appendix*, Fig. S1*B*). To reconstruct an SR SIM image frame, one needs to separate the down-modulated frequency information from the original optical transfer function (OTF) using $\phi_{n}$, shift this information to the correct location in the frequency domain using $\vec{p}$, and then, scale it to the proper power using a.

We found that, for brain tissue, deriving the illumination parameters from the raw data series of the biological sample themselves led to reconstruction artifacts and spurious spatial frequency components (*SI Appendix*, Fig. S1 *C–E*). We identified two main sources of the artifacts, both resulting from the 3D distribution of fluorescent structures typical of brain tissue. The low-frequency background from out-of-focus fluorescence leads to erroneous estimation of the illumination vector (*SI Appendix*, Fig. S3 *A* and *B*). When the fluorescent objects used for parameter evaluation are dominated by features larger than the diffraction limit, the calculated modulation depth become inaccurate (*SI Appendix*, Fig. S3 *C–E*).

Instead, a 2D sample consisting of a sparse distribution of subdiffractive-sized beads led to accurate measurement of the illumination parameters in SIM. Indeed, bead-based measurements produced consistently accurate results, whereas illumination parameters derived from a series of different brain slices did not (*SI Appendix*, Fig. S1 *F* and *G*). Therefore, at the beginning of each experiment, we measured illumination parameters from a reference bead sample (*SI Appendix*, Fig. S1*H*) and used these parameters for subsequent SIM reconstruction of brain datasets (*SI Appendix*, Figs. S1 *I* and *J* and S4*A*).

### AO Correction Is Essential for Artifact-Free SR SIM Images.

In addition to the structured illumination parameters, to reconstruct an SIM image, one also needs to know the OTF of the microscope. In ultrathin in vitro samples, such as 2D cell cultures, the OTF may be adequately approximated by that of an ideal diffraction-limited microscope in which the image formation process is free of aberrations or experimentally determined from subdiffractive beads. However, during in vivo imaging of the brain, optical aberrations are introduced in the imaging process due to the heterogeneous refractive index throughout the brain or for mice, by the refractive index mismatch at an overlying cranial window (14). Compared with diffraction-limited methods, SR methods are more sensitive to aberrations and face greater image deterioration than diffraction-limited ones for a given amount of wavefront error (15). Fortunately, however, excitation in widefield SIM is fairly insensitive to aberrations, because at any one time, it consists of only two wavevectors necessary to create a standing wave intensity pattern within the sample. Aberration-induced changes to the phase of either beam simply shift the phase of the standing wave, and changes to the direction of either wavevector simply alter its orientation. Because both the phase and orientation of the pattern can be determined empirically during SIM reconstruction, these changes do not introduce artifacts. However, the widefield detection OTF in SIM is highly aberration sensitive, particularly near the edges of the diffraction-limited passband that contribute the highest-resolution information. Maintaining accurate diffraction-limited performance in this part of the OTF is essential to avoiding artifact-producing gaps in the reconstructed SIM OTF (16) as well as artifact-producing errors in the amplitude and phase of the overlapped regions of the frequency-shifted widefield OTFs from which the SIM OTF is constructed. Here, adaptive optical correction is essential.

**Fig. 1.**
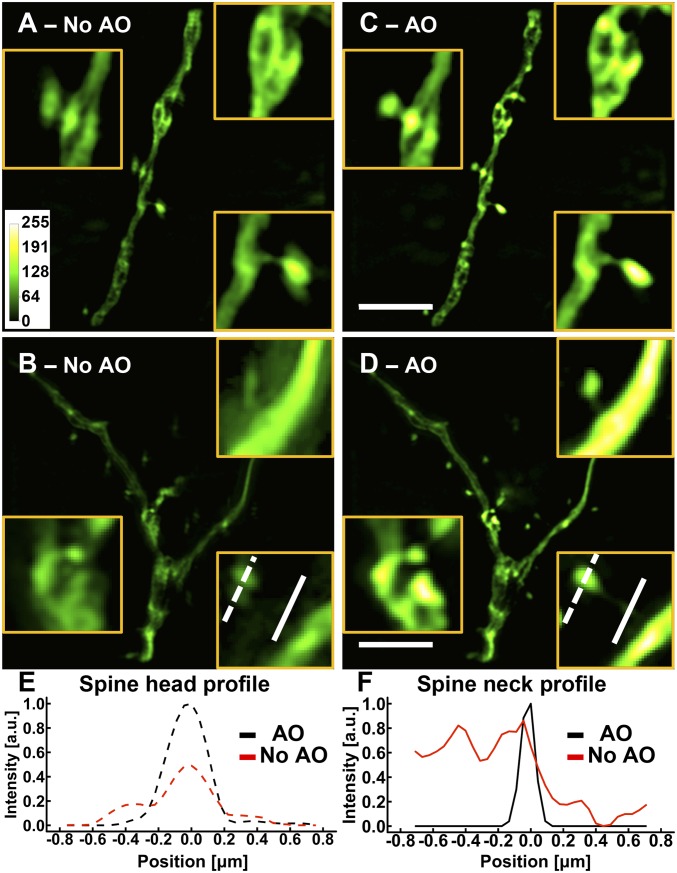
AO is essential for SIM imaging in brain tissue. (*A*–*D*) Images of dendrites at a depth of 25 μm in a cortical slice of a Thy1-GFP line M mouse (*A* and *B*) without and (*C* and *D*) with AO. (Scale bars: 5 μm; *Inset* widths: *A* and *C*, 3 μm; *B* and *D*, 2 μm.) (*E* and *F*) Line profiles of (*E*) a spine head and (*F*) a spine neck with and without AO as identified by the lines in *B* and *D*. Images were normalized to the AO condition.

To begin, we evaluated the effect on SIM of aberrations introduced by a cranial window in vivo by imaging a fixed brain slice from a Thy1-GFP line M mouse through an overlying cover glass (No. 1.5; 160- to 190-μm thick; Fisher Scientific), slightly tilted by $2^{○}$, to approximate typical in vivo imaging conditions. We used the correction collar of the imaging objective to minimize spherical aberrations as much as possible. After adjusting the correction collar, we measured a residual wavefront with an rms (σ) error of 0.11 μm via direct wavefront sensing using a two photon-induced fluorescent guide star and corresponding Shack–Hartmann wavefront sensor (17–20). Using bead-measured illumination parameters but the theoretical, aberration-free OTF for image reconstruction, we obtained SIM images of dendrites and dendritic spines where fine structural details were smeared, distorted, or split (Fig. 1 *A* and *B* and *SI Appendix*, Fig. S5).

We then corrected the residual aberrations with the deformable mirror, retook the raw data series, and reconstructed the now aberration-free images using the same illumination parameters and theoretical OTF. The resulting SIM images showed substantial improvements in brightness and spatial resolution (Fig. 1 *C* and *D*), with the improvements particularly noticeable for dendritic spines and spine necks: AO sharpened spine heads, removed artifacts, and allowed spine necks to be properly visualized (Fig. 1 *E* and *F*). Given that these small, dim structures are the ones that most benefit from SR, measuring and correcting optical aberrations with AO are essential for the optimal application of SIM in vivo.

### Assessing the SR Performance of SIM for Brain Imaging.

**Fig. 2.**
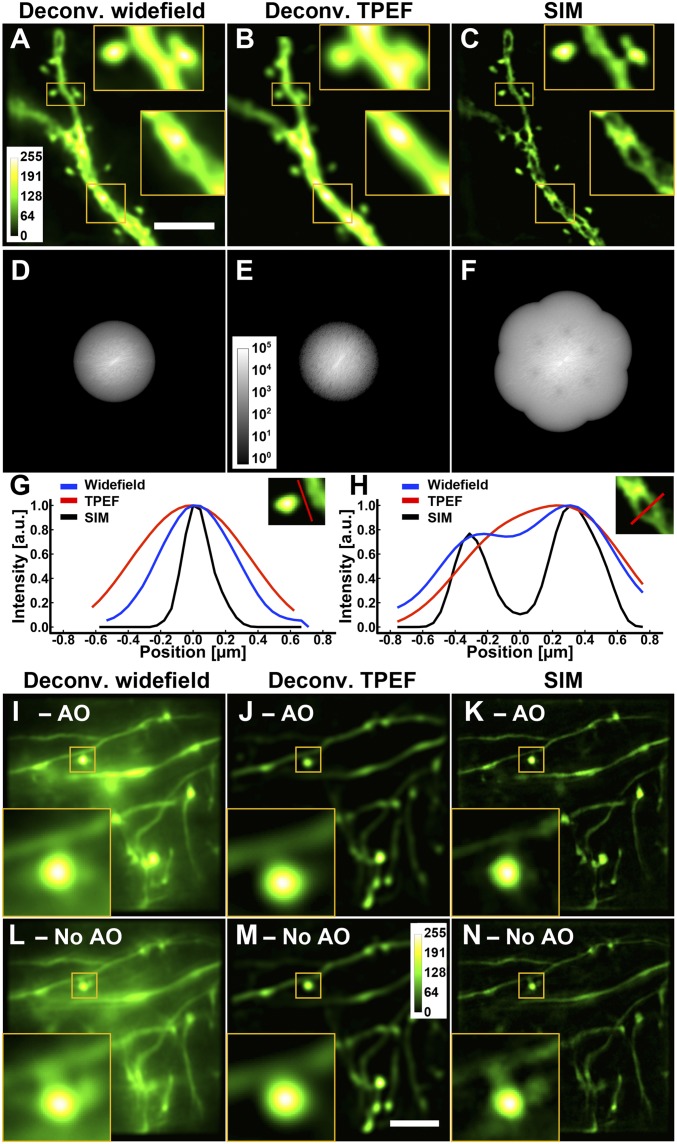
SIM yields spatial resolution superior to deconvolved widefield and TPEF microscopy both ex vivo and in vivo. (*A–F*) Images and corresponding OTFs of the same dendritic structure in a Thy1-GFP line M brain slice at a depth of 25 μm obtained with different imaging modalities, all with AO: (*A* and *D*) deconvolved widefield, (*B* and *E*) deconvolved TPEF, and (*C* and *F*) SIM. (Scale bar: 5 μm; *Inset* widths: 3 μm.) (*G* and *H*) Line profiles through a spine neck and a dendritic shaft, respectively. All deconvolutions were performed with Wiener filtering. (*I–N*) In vivo images of neurites in a larval zebrafish brain at a depth of 100 μm. Images of the same neurites obtained with (*I* and *L*) deconvolved widefield, (*J* and *M*) deconvolved TPEF, and (*K* and *N*) SIM with and without AO, respectively. Images were normalized independently. (Scale bar: 5 μm; *Inset* widths: 3 μm).

We assessed the performance of AO-corrected SIM by comparing it with aberration-corrected widefield and two-photon fluorescence excitation (TPEF) point-scanning microscopy images of the same fixed mouse brain slice (Fig. 2 *A*–*C*). Because deconvolution (by Wiener filtering) is a central part of the SIM reconstruction algorithm, to ensure a fair comparison, we also deconvolved the widefield and two-photon fluorescence images to enhance the features having high spatial frequencies. The resolution improvement was apparent in the frequency domain, with the OTF for SIM extending into higher spatial frequencies (Fig. 2 *D*–*F*) to 1.75× diffraction limit (*SI Appendix*). We further measured the lateral resolution for SIM, defined here as the FWHM of 0.1-μm-diameter fluorescence beads, and obtained a value of 190 ± 11 nm (*n* = 15), a 1.7× improvement compared with widefield microscopy and within 2% of the theoretical value (193 nm). The line intensity profiles across a spine neck (Fig. 2*G*) and a structure internal to the dendritic shaft (Fig. 2*H*) also demonstrated the superior resolving power of SIM, which reported a narrower spine neck and the exclusion of fluorescence by an organelle (most likely a mitochondrion) in the dendritic shaft.

For brain samples devoid of motion, optimizing the image acquisition and reconstruction as detailed above is sufficient for applying SIM in vivo. For example, we performed in vivo SIM imaging in the brain of an anesthetized casper larval zebrafish, where the spinal projection neurons were labeled with dextran-conjugated Alexa Fluor 488, and compared its performance with widefield and TPEF microscopy. Being relatively transparent and sparsely labeled, this specimen allowed us to acquire SIM images at a depth of 100 μm. We measured sample-induced aberrations by direct wavefront sensing from TPEF guide star and corrected them with a deformable mirror (*SI Appendix*, Fig. S5*F*). Comparing the widefield, TPEF, and SIM images (Fig. 2 *I*–*K*), we found that the SIM images have the out-of-focus signal comparatively suppressed and possess the highest spatial resolution. Furthermore, while the effect of the sample-induced aberrations on the diffraction-limited imaging modalities was minimal (Fig. 2 *L* and *M*), they resulted in substantial artifacts in the SIM images (Fig. 2*N*), further demonstrating the essential role that AO plays in SR in vivo imaging.

### Strategies to Combat Motion-Induced Artifacts for in Vivo SIM in the Mouse Brain.

In contrast to the stationary specimens of fixed brain slices or the anesthetized zebrafish larval brain imaged in vivo above, applying SIM to the brain of a live mouse raises additional challenges. For such imaging, we fix the skull rigidly in place and press the cranial window gently onto the brain to minimize brain movements caused by the animal’s motion, respiration, and heartbeat. We can further reduce such motions using light anesthesia. Even then, however, we found that residual periodic brain motion always remained in the plane parallel to the cranial window, typically with an amplitude of ∼0.1 μm and a frequency on the order of tens of hertz. This residual motion caused blurring of the image within each raw acquired frame and unanticipated phase shifts between the applied illumination pattern and the specimen. We found that both effects, when uncorrected, led to SIM images of the dendritic structures with severe artifacts when imaging the brain of an anesthetized Thy1-GFP line M mouse brain in vivo (*SI Appendix*, Fig. S6*A*).

**Fig. 3.**
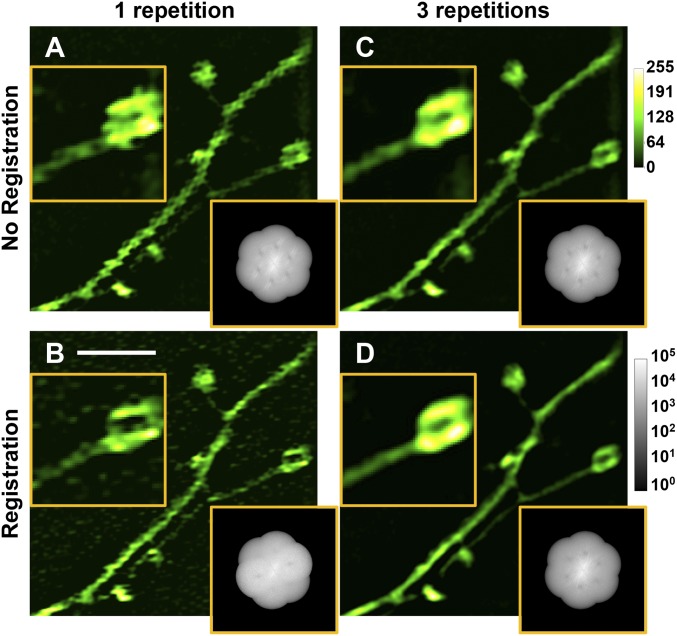
Strategies to combat motion-induced artifacts for in vivo SIM in the mouse brain. SIM images and OTFs reconstructed from raw data series (*A*) with one repetition and without raw image registration, (*B*) with one repetition and with registration, (*C*) with three repetitions and without registration, and (*D*) with three repetitions and with registration. Images were normalized independently. (Scale bar: 3 μm; *Inset* widths: 2.5 μm.)

To compensate for this motion, we implemented several approaches to optimize both image acquisition and reconstruction. First, we observed that camera integration times longer than 5 ms led to image blurring and artifactual spatial frequency components in the OTF. Therefore, we limited the exposure time to 1–2 ms and thereby, obtained higher-quality SIM images (*SI Appendix*, Fig. S6*B*). Second, to remove the frame-to-frame motion-induced image shifts, we registered the images from the raw data series to their collective averaged image with subpixel accuracy before reconstruction (21). This reduced but did not completely remove the reconstruction artifacts, because the phase of the structured illumination pattern relative to the specimen was also changed by sample motion. It was thus necessary to reevaluate the phase $\phi_{n}$ in each image of the raw data series. We chose noniterative Wicker phase estimation for our data (22). The resulting SIM image had reduced motion artifacts (compare Fig. 3 *A* and *B*) but a suboptimal signal-to-noise ratio, partly because the motion of the sample relative to the illumination caused the phase space to be undersampled. To ensure that the $\phi_{n}$ is sufficiently spaced in the phase space, we repeated data acquisition and took multiple images for each orientation and phase of the applied illumination pattern (Fig. 3 *C* and *D*). The motion of the sample then ensured that its structures experienced a sufficient diversity of $\phi_{n}$ values. In practice, we found that three repeats of data acquisition combined with image registration and noniterative Wicker phase estimation yielded SIM images of the mouse brain in vivo with minimal artifacts (Fig. 3*D* and *SI Appendix*, Fig. S6 *C* and *D*).

### In Vivo Morphological and Functional SR Imaging of the Mouse Brain.

**Fig. 4.**
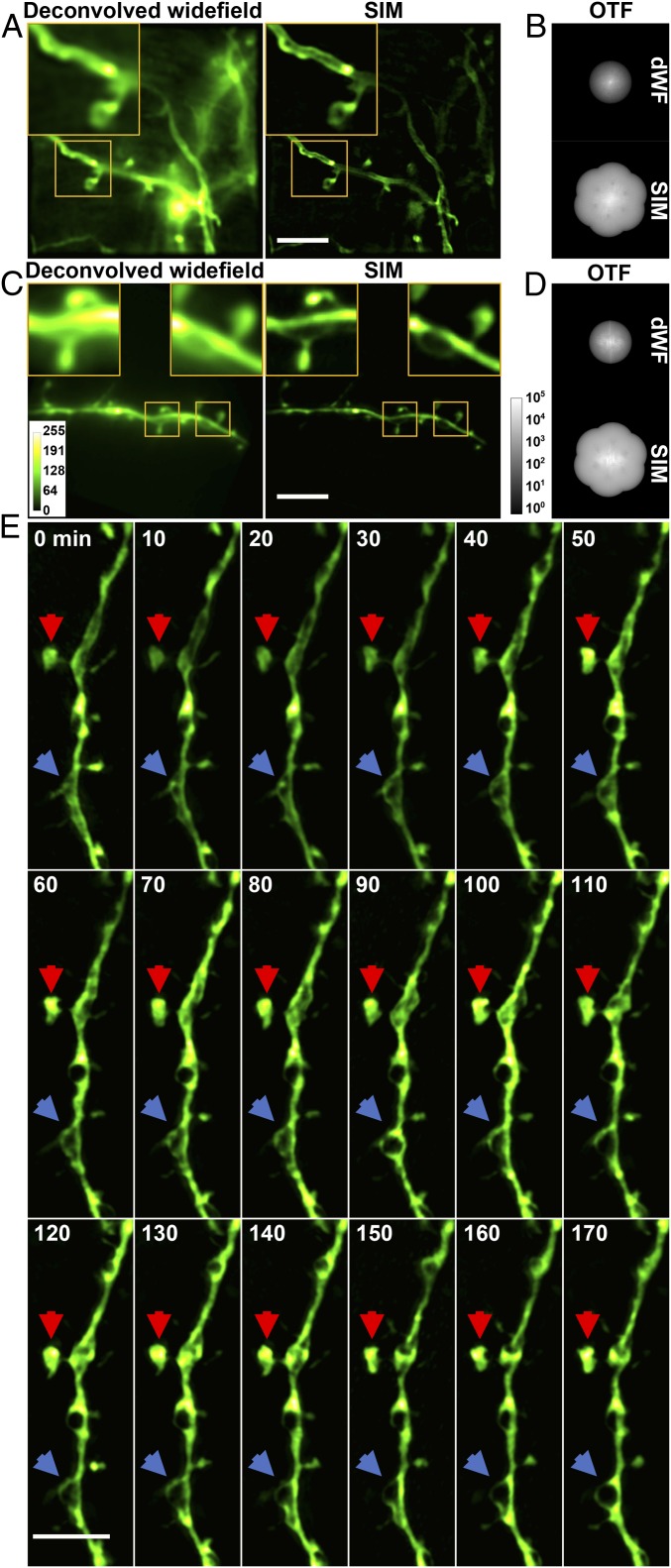
In vivo SR imaging of the mouse brain with AO SIM. (*A*) Deconvolved widefield (dWF) and SIM images of dendrites expressing ChR2-GFP, a membrane label. (Scale bar: 5 μm; *Inset* width: 5 μm.) (*B*) OTFs of the SIM and dWF images in *A*. (*C*) dWF and SIM images of neurons expressing cytosolic GFP (Thy-1 line M mouse). (Scale bar: 5 μm; *Inset* width: 3 μm.) (*D*) OTFs of the SIM and dWF images in *C*. (*E*) Time-lapse in vivo SIM images showing structural dynamics of a dendrite at a depth of 25 μm in the brain of a Thy1-GFP line M mouse after KCl injection. Arrows point to highly dynamic structures. Images were normalized independently. (Scale bar: 4 μm.)

Using these strategies, we were able to routinely image in the mouse brain in vivo at a depth of 25 μm below the dura and in some cases, at a depth of 50 μm (*SI Appendix*, Fig. S6 *C* and *D*). The improvement in spatial resolution was apparent when we compared diffraction-limited widefield images with SIM images of dendrites of neurons using either membrane or cytosolic fluorescence labels (Fig. 4 *A*–*D*). For both samples, spine necks were much better defined by SIM, and in the case of membrane labeling, only SIM was capable of detecting the separation between the opposing membranes of the dendrites.

To evaluate the performance of SIM in assessing structural changes in vivo, we injected potassium chloride (KCl; 200 nL at 50 mM) at a depth of 50 μm in the brain through an opening in the cranial window immediately before imaging. Such KCl treatment is known to cause neuronal depolarization and lead to dendritic beading (23) when mitochondria swell from an ellipsoidal to a spherical shape. Imaging the same location every 10 min for 3 h, we indeed observed beading in dendrites, with dark regions likely corresponding to swelled mitochondria (Fig. 4*E*, blue arrows). In addition, SIM enabled us to visualize the fine structural dynamics of changing shape and fluorophore distribution of the spine head (Fig. 4*E*, red arrows). By comparison, these structural changes were much less apparent in diffraction-limited widefield images (Movie S1 and *SI Appendix*, Fig. S7).

In addition to neuronal morphology, in vivo imaging serves as a powerful tool for recording neuronal activity in the brain. We, therefore, tested the efficacy of in vivo SIM for functional imaging of neurons expressing the genetically encoded calcium indicator GCaMP6 (24). Injecting bicuculline to evoke calcium activity, we found the sensitivity and speed of our method, at 9.3 SR frames per second, to be sufficient in following calcium activity in vivo (Movie S2 and *SI Appendix*, Fig. S8).

## Discussion and Conclusion

Compared with other SR imaging methods, SIM has the advantage of working with a diverse array of conventional fluorophores, including activity indicators. Using widefield detection and requiring only nine raw images to construct an SR frame (27 raw images per SR frame in vivo), it can also be performed at high speed, enabling the monitoring of fast dynamic processes at high resolution. To apply it in vivo, we devised a series of approaches ranging from fast data collection to image reconstruction and phase estimation to address the challenges arising from imaging the brain, which is optically heterogeneous, spatially complex in three dimensions, and often, constantly moving in live animals. We operated SR SIM in a regime capable of optical sectioning to suppress contributions from out-of-focus fluorescence. We chose a grating spatial frequency corresponding to 75% of the full N.A. to obtain an optical sectioning depth of 0.45 μm. We further used the OTF attenuation technique (25, 26), where frequency components corresponding to the original and shifted zero-frequency bands were suppressed with a Gaussian notch filter (*SI Appendix*). OTF attenuation improved the quality of SIM images not only by rejecting the out-of-focus signal from the SR image, but also by eliminating the periodic reconstruction artifacts caused be the out-of-focus signal shifted to high spatial frequency (*SI Appendix*, Fig. S9). Due to the 3D morphology of neuronal processes, this approach is necessary even when imaging sparsely labeled samples, as were most samples used in our study. In samples with much denser fluorescent labeling, these approaches become less effective due to the irrepressible nature of photon noise.

Also essential to applying SIM successfully in the brain was our use of AO. Most often applied in conjunction with TPEF microscopy for brain imaging (14, 19, 20, 27), AO has also been implemented with other SR imaging modalities, including stimulated emission depletion (15, 28) as well as single-photon (16, 29) and multiphoton versions of SIM (30). Here, we used an AO approach based on a nonlinear fluorescent guide star and direct wavefront sensing (17–19) for single-photon linear SIM. We found that even very small wavefront aberrations, which had minimal impact on the diffraction-limited imaging modalities (such as TPEF microscopy), can lead to severe imaging artifacts in SIM. Given that other SR modalities potentially offer even higher resolution, the use of AO is likely even more critical to achieve artifact-free extended resolution in the brain by any such modality.

With these optimizations, we were able to routinely image sparsely labeled neural structures in vivo using SIM. For optically scattering samples, such as the live mouse brain, the image depth is currently limited by the signal-to-background ratio to ∼50 μm for green fluorophores. Fluorescent dyes emitting at longer wavelengths can be expected to reduce the scattering background and allow SIM imaging at greater depths.

In summary, we systematically addressed the challenges specific to in vivo SR imaging with SIM. Although we focused on in vivo brain imaging, the strategies developed here can be applied to optically heterogeneous samples with 3D fluorescence labeling in general.

## Materials and Methods

All experiments involving animals were conducted according to the NIH guidelines for animal research and were approved by the Institutional Animal Care and Use Committee at Janelia Research Campus, Howard Hughes Medical Institute. Detailed materials and methods are available in *SI Appendix*.

## Supplementary Material

Supplementary File

Supplementary File

Supplementary File
