# Supplementary material for: Dynamic super-resolution structured illumination imaging in the living brain
Source: Proc Natl Acad Sci U S A. 2019 Apr 26;116(19):9586–91. doi: 10.1073/pnas.1819965116 (PMC6511017; doi:10.1073/pnas.1819965116)
Supplement: Supplementary File [file pnas.1819965116.sapp.pdf]

PNAS
www.pnas.org

Supplementary Information for

Dynamic super-resolution structured illumination imaging in the living brain

Raphaël Turcotte, Yajie Liang, Masashi Tanimoto, Qinrong Zhang, Ziwei Li, Minoru Koyama, Eric Betzig and Na Ji

Eric Betzig and Na Ji.
E-mail: betzige@janelia.hhmi.org, jina@berkeley.edu

This PDF file includes:

Supplementary text
Figs. S1 to S10
Captions for Movies S1 to S2
References for SI reference citations

Other supplementary materials for this manuscript include the following:

Movies S1 to S2

www.pnas.org/cgi/doi/10.1073/pnas.1819965116

Supporting Information Text

Supplementary Materials and Methods

AO-SIM optical setup. A SIM pathway was added to an optical setup that was used for direct wavefront sensing and AO correction of TPEF microscopy (SI Appendix, Fig. S2) as described previously (1–3). Both the TPEF microscope and direct wavefront sensing system shared the same excitation path and used a near-infrared femtosecond pulsed laser (920 nm, Coherent, Chameleon Ultra II) for multiphoton fluorescence excitation. After a 10× expansion by a pair of lenses (focal lengths (FL): 50 mm and 500 mm), the excitation beam was incident on a deformable mirror (DM; Alpao, DM 97-15). The DM was conjugated to a pair of galvanometer mirror (Cambridge Technology, 6215H) and the objective lens back focal plane (Nikon, CFI Apo LWD 25XW, 1.1 NA and 2 mm WD) with achromatic lens pairs (FL from DM to objective: 300 mm, 100 mm, 85 mm, 85 mm, 100 mm, and 400 mm). The same objective was used for focusing the excitation light and collecting the emitted fluorescence. A dichroic mirror (D1 in SI Appendix, Fig. S2, Semrock, FF665-Di02-25x36) reflected the emitted fluorescence, which was then focused by a lens (FL: 75 mm) and spectrally filtered (Semrock, FF03-525/50-25 for brain imaging) before being detected by a photomultiplier tube (PMT, Hamamatsu, H7422-40) for TPEF imaging. For wavefront sensing, the dichroic D1 was replaced (Semrock, FF409-Di03-25x36) for the emitted fluorescence to be descanned and reflected by dichroic D2 (Semrock, FF875-Di01-25x36). The light was then relayed to a Shack–Hartmann (SH) wavefront sensor by a pair of lenses (FL: 100 mm and 150 mm). The SH sensor was composed of a lenslet array (Edmund Optics, 64-483) and a camera (Andor iXon3 897 EMCCD) at its focal plane. For AO, the wavefront aberrations were measured from the shift of the spots in the SH pattern. The first 55 modes of the Zernike polynomial were used for calibration and wavefront decomposition (2). The corrective pattern was then applied to the DM. System aberration correction was carried out before all experiments.

For SIM pathway, output from a 488 nm continuous-wave laser (Coherent, Sapphire 488LP, 100 mW) was expanded (Thorlabs, GBE02-A) after passing through an acousto-optic tunable filter (AOTF; AA Quanta Tech, AOTFnC-400.650-TN). The beam then propagates through a polarizing beam splitter (Thorlabs, PBS251), an achromatic half-wave plate (HWP; Bolder Vision Optik, BVO AHWP3), and a ferroelectric spatial light modulator (SLM; Forth Dimension Displays, SXGA-3DM). The polarization of the diffracted light was controlled by an achromatic quarter-wave plate (QWP; Bolder Vision Optik, BVO AQWP3) and a second HWP to ensure maximum interferences at the sample, both mounted in fast rotators (FR, Finger Lakes Instrumentation, A24021). The SLM was positioned at one focal length away from a lens (FL: 400 mm) for conjugation with the sample plane. A mask was placed at the other focus to let through the two 1st-order diffraction beams and block the 0th-order diffraction and the spurious diffraction due to the SLM being pixelated. The mask was imaged onto the DM by a pair of lenses (FL: 200 mm and 300 mm) and the objective back focal plane, and inserted into the pulsed laser path by moving a mirror (D3) mounted on a translation stage (SmartAct, SLC-24150-D-L-BK). The interference of the two diffracted beams at the objective focus generated a harmonic patterned illumination.

The SLM displayed binary grating images, thus controlled the period, orientation, and phase of the harmonic pattern at the sample. Image series for SIM were acquired at 3 equidistant orientations. At each orientation, 3 equidistant phases covering a full illumination period were applied. When motion was present, multiple images were recorded at each phase. The frequency of the grating pattern was chosen to be 75% of the diffraction limit cutoff for sufficient rejection of the out-of-focus fluorescence background while maximizing the resolution gain. The emitted fluorescence was collected by the objective, separated from the illumination light by a dichroic (D4; Chroma, ZT405/488/560tpc, 22.5deg) and imaged (FL: 175 mm) onto a sCMOS camera (Hamamatsu, Orca Flash 4.0 v2 sCMOS) after being spectrally filtered (Semrock, Di01-R488/561-25x36, FF03-525/50-25). The imaged pixel size at the camera was 89 nm. A restricted area of 256×256 pixels^2 was captured to achieve millisecond frame-rate. For SIM imaging mode, D1 and D2 were changed to long-pass dichroic mirrors (Semrock, FF409-Di03-25x36). Volumetric data was acquired by translating the objective lens with its piezo-electric mount (Physik Instrumente, P-726.1CD). Experimental settings for all images presented are provided in detail (SI Appendix, Fig. S9).

Minimization of aberrations via correction collar and adaptive optics. Optical aberrations encountered for *in vivo* imaging of the mouse brain were minimized in two steps. First, the positioning of the mouse brain was optimized with direct wavefront sensing. Direct wavefront sensing was implemented as a Shack-Hartmann wavefront sensor of the multiphoton fluorescence signal positioned at a descanned conjugated pupil plane. For this step, the wavefront was measured immediately below the cranial window and decomposed into Zernike coefficients. We then iteratively adjusted the objective correction collar and the tilt of the brain to minimize spherical aberrations and coma introduced by the cranial window, respectively (3). Images obtained under this condition is considered as “No AO”. Images taken under the “AO” condition had the residual wavefront error corrected with the deformable mirror.

SIM reconstruction algorithm. The raw data series with structured illumination were reconstructed into the super-resolution 2D linear SIM images using a custom algorithm. Our algorithm was based on diverse aspects of previously published strategies (Wiener deconvolution (4), illumination parameter estimation (5, 6), non-iterative Wicker phase estimation (7), ungrading (8), OTF attenuation (9, 10), the open-source FairSIM library (11)), and thus did not closely follow existing reconstruction algorithms. We therefore provide detailed flowcharts for our algorithms with and without the corrections for sample motion in SI Appendix, Fig. S4. Briefly, each raw image was pre-processed in the real space by subtracting the background using a rolling-ball algorithm (256 pixels in diameter) and by attenuating the edges with a $sin^2(x)$ function of 10-pixel half-period. If motion correction was required, images were registered to the average image of the series using cross-correlation and rigid translation. Illumination parameters were estimated once per experimental session using data obtained from a 2D sample of

0.1-μm beads as the reference. The parameters were then used to separate the positive and negative 1st-order bands from the 0th one in the Fourier domain for each orientation of the patterned illumination. After separation, the first-order bands were shifted to their appropriate location using the illumination vector $\vec{p}$ and a complex scaling was applied using the modulation depth a and phase ϕ_n information. At this point, the out-of-focus information was filtered out using the OTF-attenuation technique with a Gaussian notch filter (depth: 100%, full width at half maximum (FWHM): 5 cycles/μm) (9, 10). This operation was implemented simultaneously to the ungrading (Wiener filtering without normalization) on individual bands (8). The bands were then added together for all the orientations of the patterned illumination. The Wiener denominator was applied only after this summation. Finally, the data was apodized with a power function at the cutoff frequency and Fourier-transformed to obtain the spatial domain super-resolution SIM image. For volumetric data, each axial plane was reconstructed independently.

Evaluating the illumination parameters. Illumination parameters (the illumination vector $\vec{p}$, the modulation depth a, and the phase (ϕ)) were evaluated from raw image series of the reference bead samples. Two phase variables should be defined: 1) the global phase offset (ϕ_{global}), from the assumption of equidistant phase steps, describes the offset for all the phases for a given orientation of the illumination pattern, and 2) the phase (ϕ_n) is for an individual raw image. Following the pre-processing described above, bands were separated assuming equi-distant phase step. The illumination vector was first determined by finding the maximum of the complex cross-correlation of the overlap region between the 0th and non-shifted $+1^{st}$ bands. The coarse estimation was further refined to subpixel accuracy by maximizing the complex cross-correlation between the 0th and shifted $+1^{st}$ band with parabolic interpolation. This latter step was repeated 3 times. The cross-correlation for both the coarse and fine estimation of $\vec{p}$ was done on bands that were OTF-corrected (multiplied by the complex conjugated of the OTF) to minimize the noise contribution. For samples with no motion, a linear cross-correlation was then performed to estimate the modulation depth a and the global phase offset (ϕ_{global}). When necessary, the phase (ϕ_n) for each image was further refined with non-iterative Wicker phase estimation (7). If no sample motion was present, this step was not essential and had no visible impact on the final images. Finally, these parameters were applied to separate the bands for brain data. For samples with apparent motion, registering the background-corrected images was necessary, and so was evaluating the phases (ϕ_n) for each image from brain data with non-iterative Wicker phase estimation. Also, if repeated image acquisition was applied, a had to be estimated on beads from the bands newly separated with the updated phase information.

Mouse preparation. All experiments involving animals were conducted according to the National Institutes of Health (NIH) guidelines for animal research and were approved by the Institutional Animal Care and Use Committee at Janelia Research Campus, Howard Hughes Medical Institute. All mice (Thy1-GFP line M or C57Bl6/J) were at least 8-week old at the time of cranial window installation. *In vivo* imaging was performed on mice under isoflurane anesthesia (~1.0% by volume in O_2) at least 2 weeks after cranial window installation.

Cranial window installation. Under isoflurane anesthesia (~1.5% by volume in O_2) and following aseptic techniques, a craniotomy of 3.5 mm in diameter was made over the left cortex. The dura was left intact. The hat-shaped cranial window, consisting of a 3.5 mm disk and a donut-like ring with 3.0-mm inner diameter and 4.5-mm outer diameter, both made from coverglass (Fisher Scientific, No. 1.5, 160-190 μm thick), was prepared by applying a UV adhesive between the two components and curing it with a UV lamp. The disk was embedded into the craniotomy and the window fixed in place with cyanoacrylate glue and dental acrylic applied on the ring. In order to keep the head stable, a titanium head-post was attached to the skull with cyanoacrylate glue and dental acrylic. For membrane imaging experiments, C57Bl/6 mice were injected during cranial window installation with viral vectors (AAV2/1-FLEX-ChR2-GFP and AAV2/1-SYN-CRE) into layer 4 of the primary visual cortex (30 nL, 500 μm below dura) with a glass pipette with a 20 μm opening beveled at 45°and back-filled with mineral oil. For calcium imaging experiments, the same procedure was followed but mice were injected with AAV2/1-SYN-FLEX-GCaMP6s and AAV2/1-SYN-CRE. Imaging was performed at least 3 weeks following viral vectors injection.

Potassium chloride and bicuculline injection into mouse cortex. For potassium chloride (KCl, Sigma-Aldrich, No. 7447-40-7) injection, the window was installed as described above, except that it consisted of a disk of 3.0-mm diameter with a 0.1-0.2 mm opening approximately 1.0 mm away from the center (drilled by a laser cutter, Universal Laser Systems, PLS6.75). The opening was sealed with silicone elastomer (WPI, Kwik-Cast sealant) prior to experiment. KCl was diluted to 50 mM in sterile buffered saline solution and loaded into a glass micropipette. An injector attached to a syringe and controlled by a syringe pump was used to administer 200 nL of the compound intraparenchymally at a depth of 50 μm. The animal was imaged immediately after injection at a location ~1 mm away from the injection site. Imaging was also performed every 5 min for 10 min prior to KCl injection and only minor structural changes, consistent with organelle motion, were observed. The same procedure was followed for bicuculline injection (TOCRIS, No. 0130) for which 200 nL of a solution at 500 μM bicuculline in sterile buffered saline solution was administered.

Fixed mouse brain slices. Mice were completely sedated with isoflurane before being transcardially perfused with 10 mL of phosphate-buffered saline (PBS), followed by 50 mL of paraformaldehyde (PFA) at 4%. After perfusion, the brain was dissected and post-fix in 4% PFA for 24 hrs at 40°C, then washed with PBS 3 times. The brain was embedded in 5% agarose in PBS, then cut on a vibratome (Leica Vibratome 1200) at a thickness of 100 μm. Sections were directly mounted on slides for drying (24 hrs), then were rehydrated with PBS before being mounted and covered with Vectashield Hardset™ (H-1400). A coverglass

(Fisher Scientific, No. 1.5, 160-190 μm thick) was also placed on top. Brain slice samples were placed on a goniometer platform (Thorlabs, GNL10) for imaging and were orthogonal to the objective optical axis unless otherwise mentioned.

Zebrafish. Zebrafish larvae of a pigmentation mutant, *casper*, were used. At 5 days postfertilization, larvae were briefly anesthetized by 0.02% tricaine (MS-222, Ethyl 3-aminobenzoate methanesulfonate) in fish system water. A fine-tip tungsten needle was dipped in fluorescent dye (20% weight/volume) of Alexa Fluor 488 conjugated with dextran, MW 10,000 (Thermo Fisher Scientific), and the fluorescent dye was injected into larvae by the needle after removing the excessive anesthetic solution. Injection sites were located in the spinal cord at the 10th spinal segment to label spinal projection neurons. After overnight incubation in fish system water, larvae were anesthetized by 0.02% tricaine, embedded in 1.6% (weight/volume) low-melting-point agar and the agar covering the imaging target was removed by a pair of fine forceps.

Beads. Fluorescent beads (Invitrogen, FluoSphere™ carboxylate-modified microsphere, yellow-green, 505/515) of 0.1 μm in diameter were used for illumination parameter estimation. The stock solution was diluted to 1:7000 in deionized water and 2 μL of the dilution was added on a microscope slide covered with a thin layer of poly-L-lysine hydrobromide (Sigma, P1399). 1.0-μm beads were prepared in the same manner, but at a 1:100 dilution in deionized water. When putting a coverglass (Fisher Scientific, No. 1.5, 160-190 μm thick) over a bead sample, a drop of water was added on the beads to avoid air gaps. Bead samples were placed on a goniometer platform (Thorlabs, GNL10) for imaging and were flat unless otherwise mentioned.

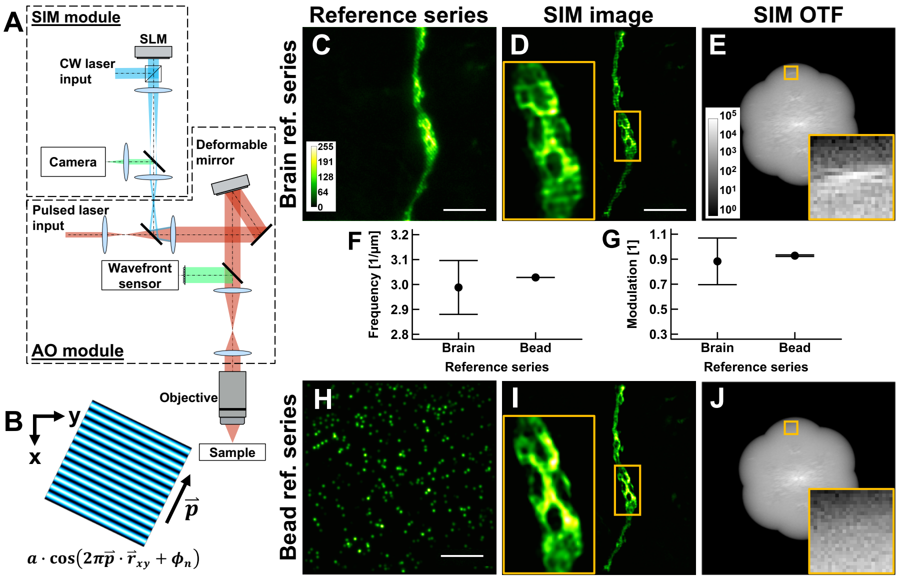

Fig. S1. A structured illumination microscope with an adaptive optics module requires accurate evaluation of illumination parameters. (A) Schematic of the microscope system. (B) An example harmonic illumination pattern and its parameters: modulation depth (a), phase (ϕ_n) for image n, and pattern frequency and direction ($\vec{p}$). (C) Example frame from a raw SIM data series of a fixed Thy1-GFP line M mouse brain slice. (D) SIM image reconstructed of the raw data series using the illumination parameters obtained from the same data, showing a periodic artifact. (E) OTF of the SIM image in (D), with inset showing a hot spot at the spatial frequency of the artifact. (F) and (G) Estimation of the illumination frequency and modulation depth from brain or subdiffraction-limited-bead raw data series, respectively. (H) An example frame from a raw data series of subdiffraction-limited beads. (I) SIM image reconstructed of the raw data series in (C) using the illumination parameters obtained from the data in (H), showing no periodic artifact. (J) OTF of the SIM image in (I), with inset showing no hot spot at the same spatial frequency as in (E). Scale bars: 5 μm; inset width in (D) and (I): 3.2 μm.

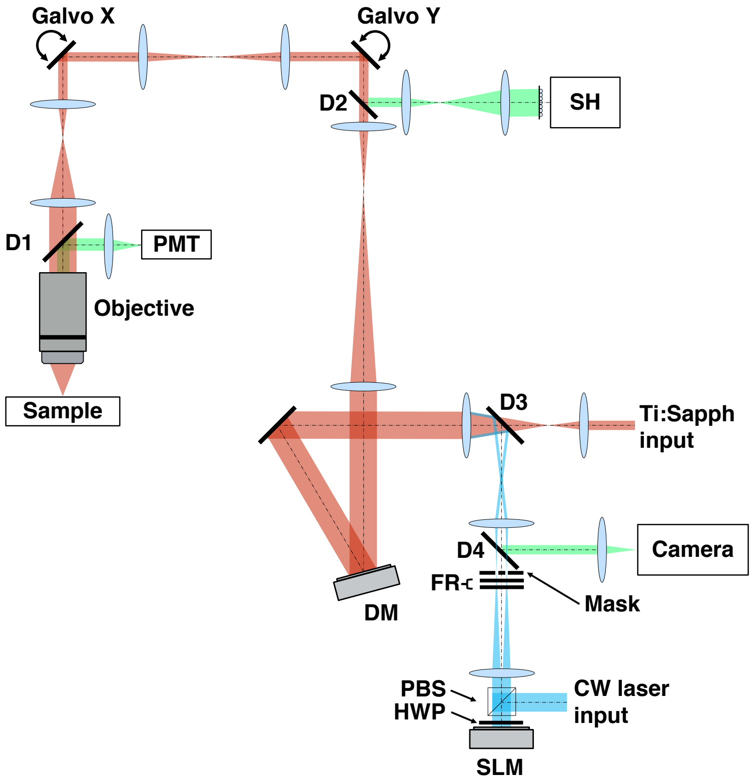

Fig. S2. Detailed optical layout of the AO-SIM setup.

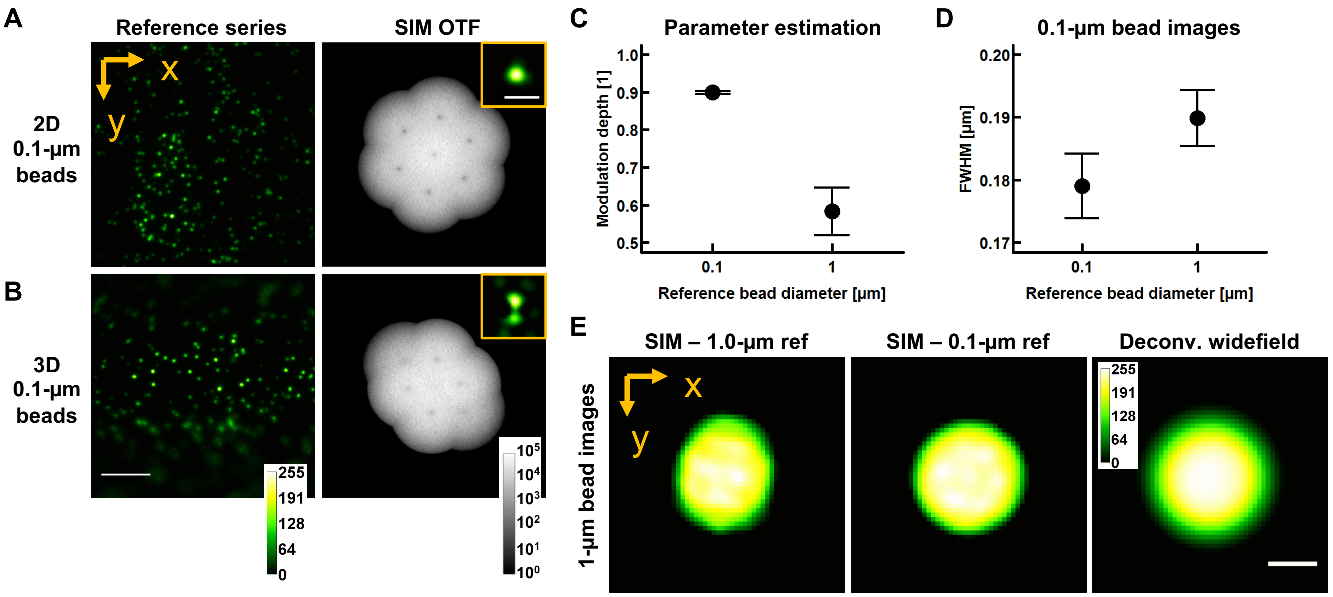

Fig. S3. Evaluating illumination parameters for structured illumination microscopy (SIM) in brain tissue. (A,B) Effects of out-of-focus signal for a sample consisting of sparse 0.1-μm-diameter fluorescent beads on a glass cover slip. (A) (Left) An example image from a SIM raw data series for a 2D bead sample that was carefully aligned, so that all the beads were in focus simultaneously, and (Right) the OTF of the reconstructed SIM image using illumination parameters derived from the raw data series. We obtained an accurate estimate of the illumination periods and orientations, leading to a SIM OTF closely resembling that predicted by theory and SR images of beads that were symmetric and artifact-free. (B) (Left) An example image from a SIM sequence for a 0.1-μm bead sample that was tilted at 6° to generate a 3D fluorescence distribution and introduced out-of-focus background, and (Right) the OTF of the reconstructed SIM image of the tilted bead sample using illumination parameters derived from the raw data series obtained from this tilted sample. Substantial errors were introduced in the SIM reconstruction, yielding artifacts such as images of split beads. Insets: example SIM images of a single bead (scale bar: 1 μm). Scale bar: 5 μm. (C-E) Effects of object size distribution. The illumination parameters were evaluated from two, optimally in focus, reference samples: 0.1-μm beads and 1.0-μm beads. (C) The illumination modulation depth derived from images of the two reference samples. Larger beads led to under-estimation of the modulation depth. (D) Lateral FWHM of 0.1-μm beads measured from SIM images reconstructed using the reference data series from the two reference samples. (E) 1.0-μm bead images from (left) a SIM reconstruction with the 1.0-μm reference, (middle) a SIM reconstruction with the 0.1-μm reference, and (right) a deconvolution of the widefield image (scale bar: 0.5 μm, images were normalized independently). Using the illumination parameters derived from 1.0-μm instead of 0.1-μm beads to reconstruct images, we obtained larger FWHMs for 0.1-μm beads and asymmetric images for 1.0-μm beads whereas symmetric profiles were expected from the deconvolved widefield image.

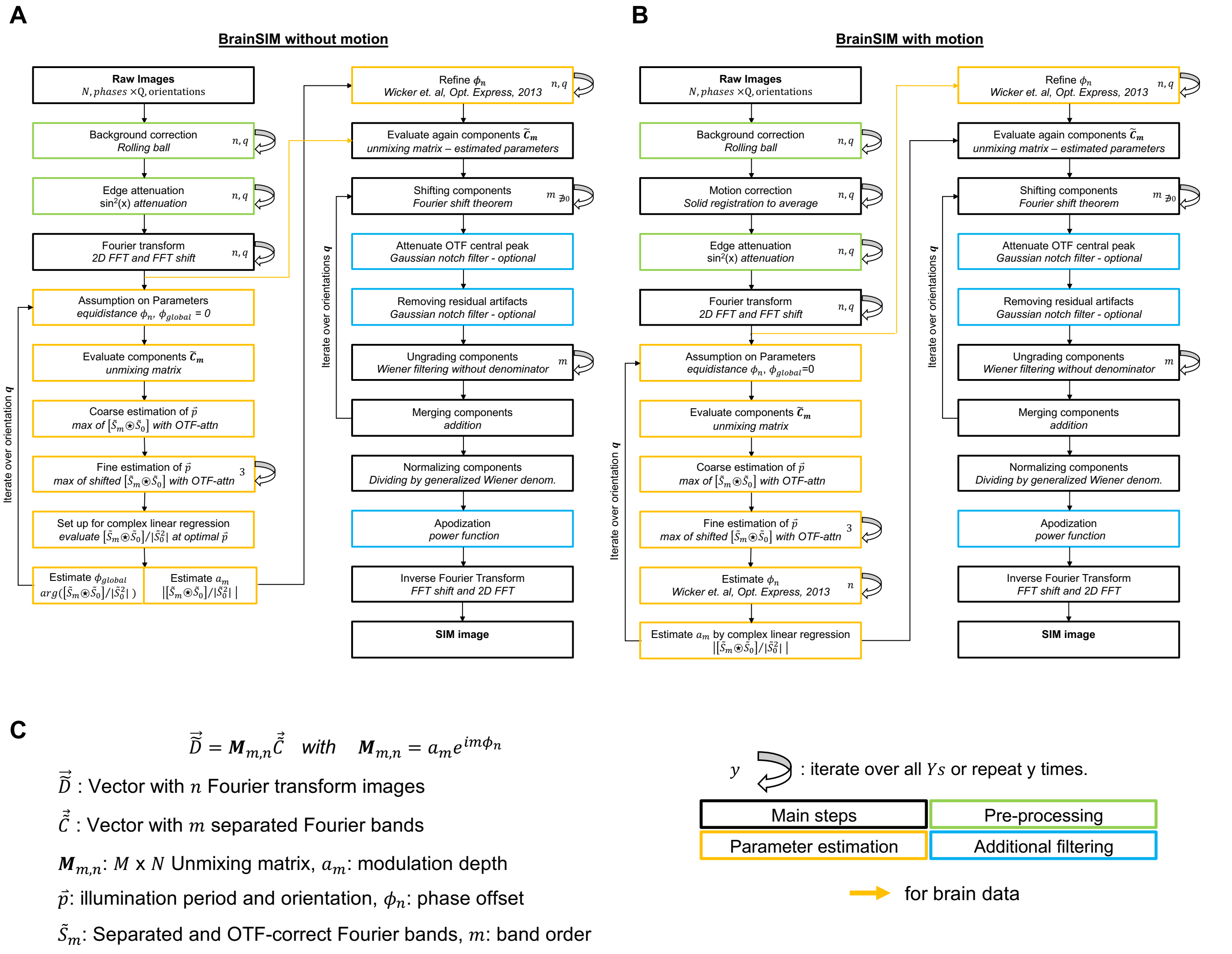

Fig. S4. Reconstruction algorithms, BrainSIM, for *in vivo* super-resolution SIM images. (A) Algorithm for raw data series in which no sample motion was present. (B) Algorithm for raw data series in which sample motion was present. (C) Definition of parameters and symbols for (A) and (B).

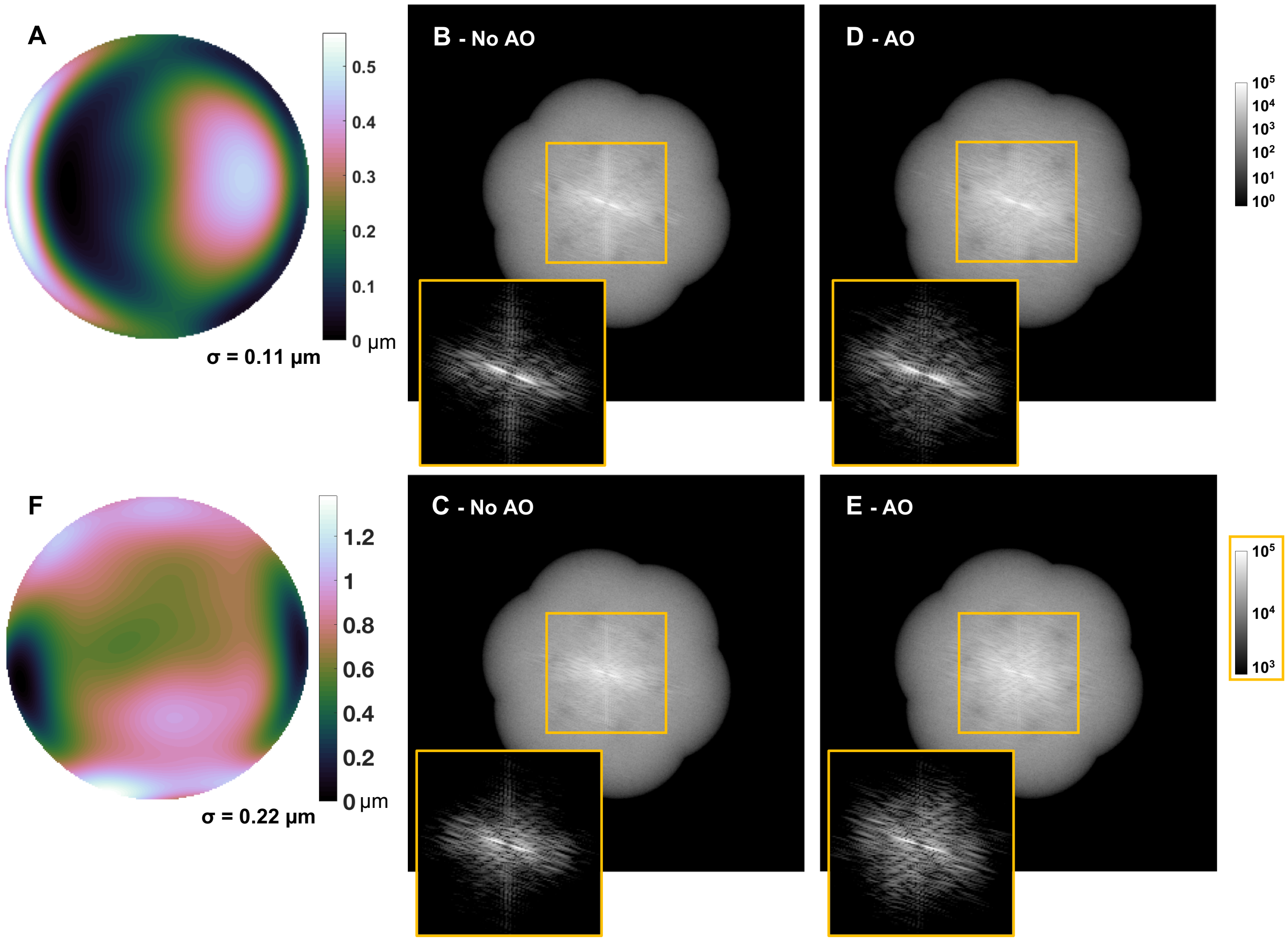

Fig. S5. Even small aberrations impact SIM image quality and OTF. (A) Corrective wavefront for the data shown in Fig. 1. (B,C) OTFs of the SIM images without AO in Fig. 1A,B, respectively. (D,E) OTF of the aberration-free SIM images with AO in Fig. 1C,D, respectively. Insets: Contrast enhanced OTFs. (F) Corrective wavefront for images in Fig. 2I-K.

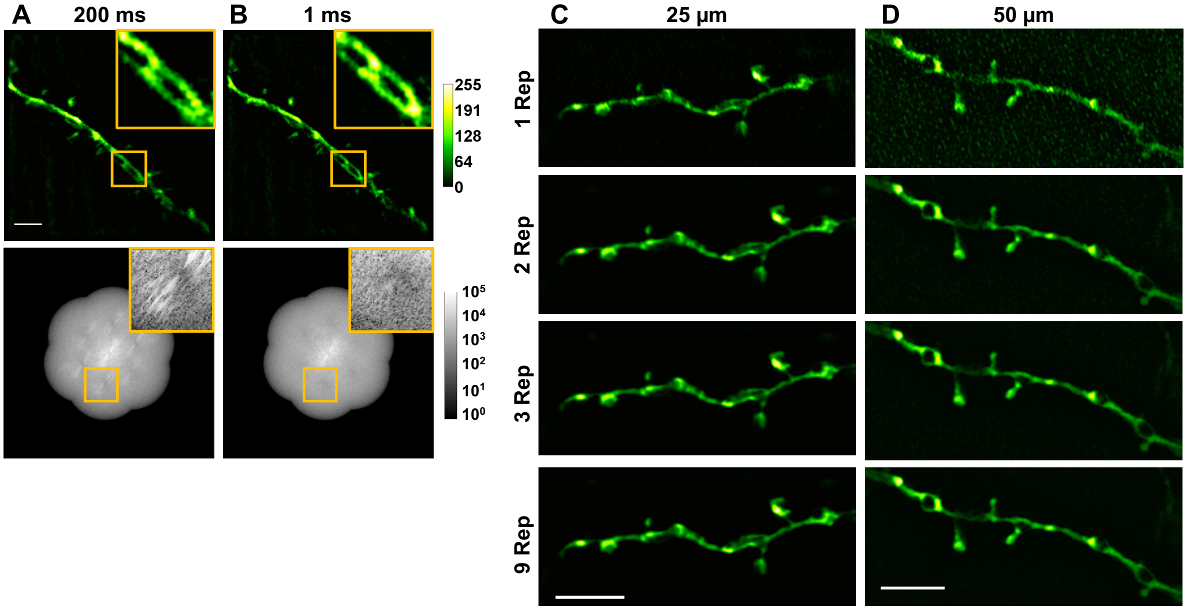

Fig. S6. Super-resolution SIM images of the *in vivo* mouse brain. (A,B) Short camera integration time reduces motion artifacts. (Top) SIM images and (bottom) their associated OTFs from raw data acquired with an integration time of (A) 200 and (B) 1 ms. Scale bar: 3 μm; inset width: 3.6 μm. (C,D) *in vivo* SIM images at depths of (C) 25 μm and (D) 50 μm for different numbers of repetitions: 1, 2, 3, and 9 repetitions (scale bar: 5 μm). Images were normalized independently. Most of the improvement in image quality is obtained when going from 1 to 2 repetitions. Occasionally artifacts are still present with 2 repetitions, thus 3 repetitions are therefore necessary.

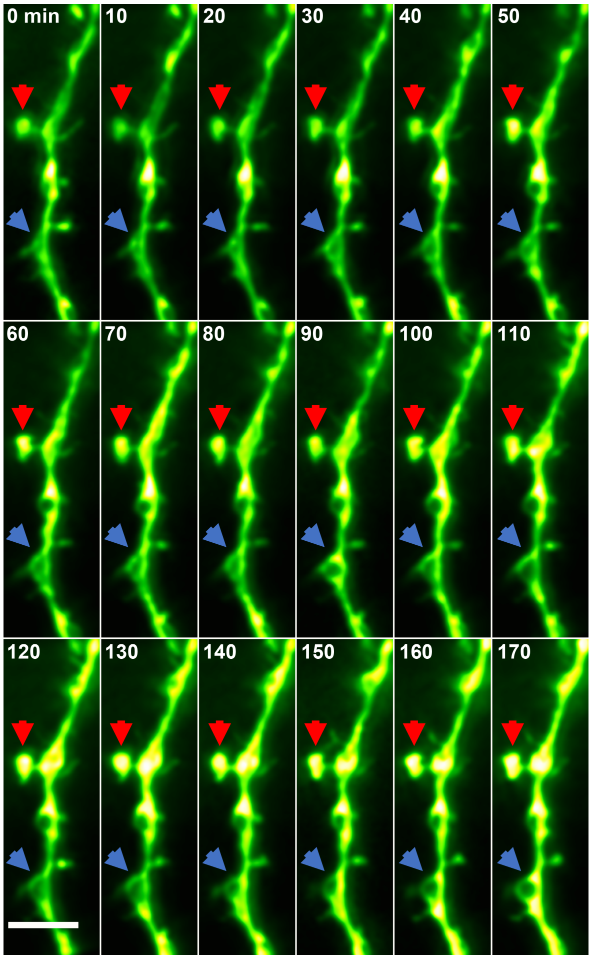

Fig. S7. Time-lapse *in vivo* deconvolved widefield images of the same dendrite as in Fig. 4 in a Thy1-GFP line M mouse following KCl injection. Scale bar: 4 μm. Images were normalized independently.

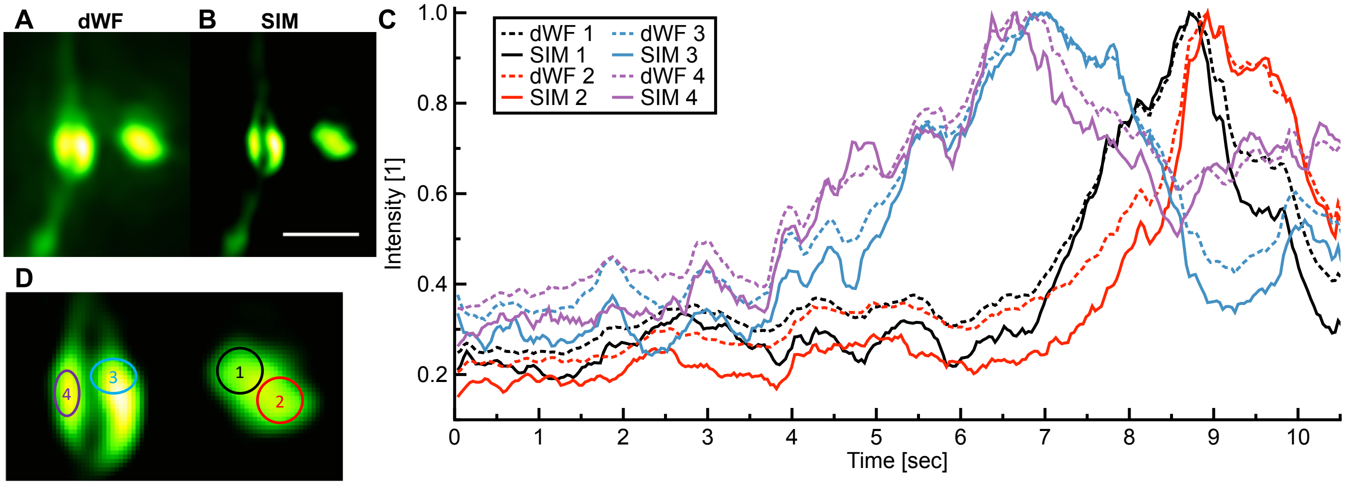

Fig. S8. Functional calcium imaging. (A) Deconvolved widefield (dWF) and (B) SIM image at a single axial plane of a putative axon and its bouton expressing GCaMP6s. Scale bar: 2 μm. (C) Time traces of the fluorescence variation after bicuculline injection in regions of interest as indicated in (D) from (A,B) (see also Movie S2).

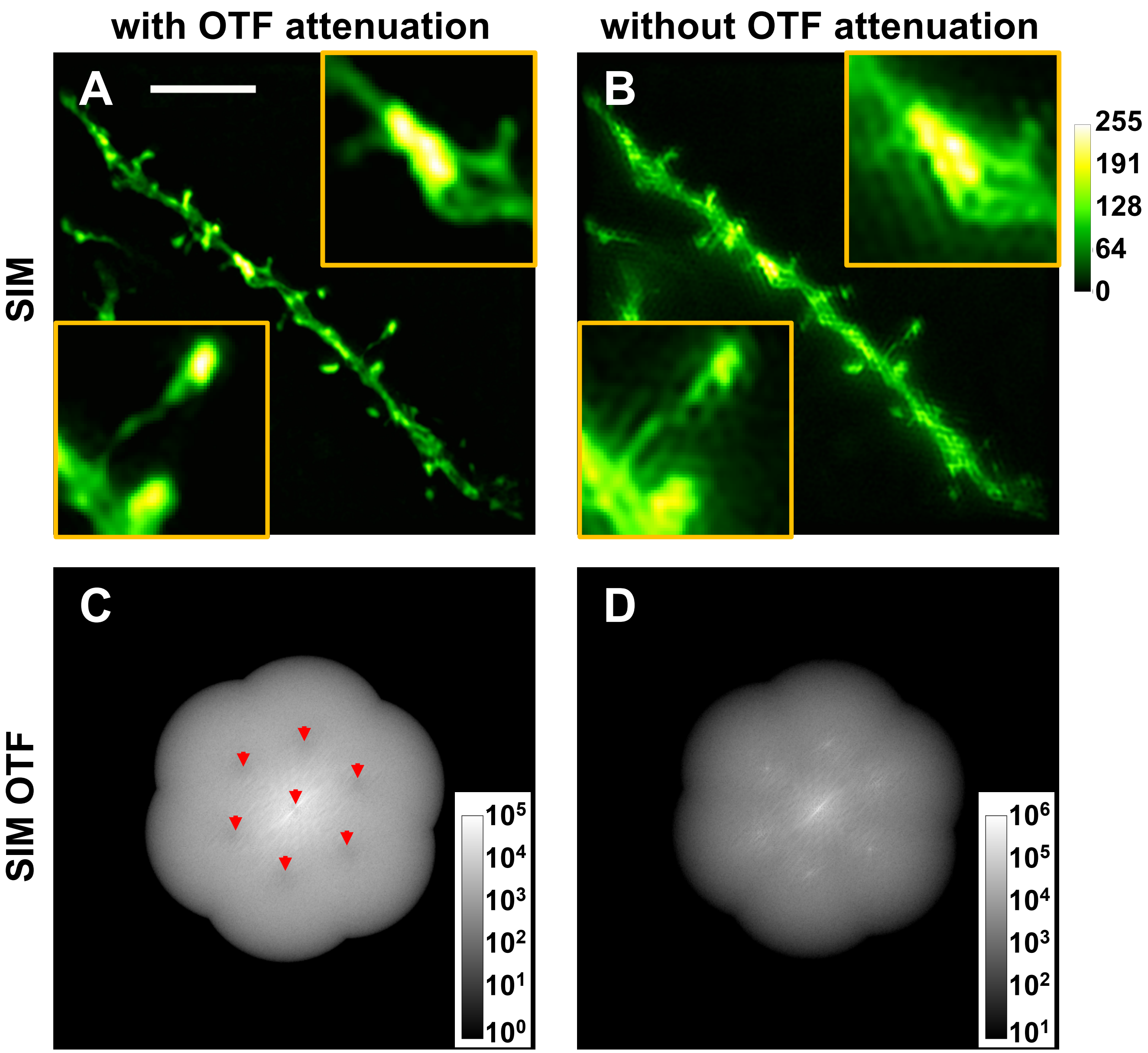

Fig. S9. OTF attenuation suppresses out-of-focus fluorescence and improve image reconstruction. SR SIM images (A) with and (B) without OTF attenuation in a fixed Thy-1 GFP line M brain slice and (C,D) their corresponding OTFs. Arrows in B point to the locations where Gaussian notch filters (depth: 100%, FWHM: 5 cycles/μm) were applied. Scale bar: 5 μm; inset width: 3 μm.

Parameters	Fig S2D,I	Fig 1A	Fig 1B	Fig 1C	Fig 1D	Fig 2A	Fig 2B	Fig 2C	Fig S9A,B	Fig 2I	Fig 2L	Fig 2J	Fig 2M	Fig 2K	Fig 2N
**Sample**	Mouse: Thy1-GFP line M, *ex vivo*									Zebrafish, *in vivo*					
**Sample prep.**	100-µm fixed brain slices under a no. 1.5 coverglass									Brain, dorsal side up					
**Imaging depth**	25 µm									100 µm					
**Modality**	Linear 2D SIM (SIM)					Widefield	TPEF scanning	SIM		Widefield		TPEF scanning		SIM	
**Illum. wavelength**	488 nm					488 nm	920 nm	488 nm	488 nm	488 nm		920 nm		488 nm	
**Illum. power**	33 W/cm^2	10 W/cm^2				24 W/cm^2	2.3 mW	24 W/cm^2	75 W/cm^2	2 W/cm^2		2.3 mW		2 W/cm^2	
**Illum. time**	50 ms	10 ms				10 ms	15 µs/pixel	10 ms	2 ms	5 ms		15 µs/pixel		5 ms	
**Axial step**	0.1 µm	0.2 µm				0.1 µm			0.4 µm	0.1 µm					
**Axial projection**	Average intensity (A.I.) over 1.0 µm					A.I. over 1.1 µm			A.I. 2.4 µm	A.I. over 2.5 µm					
**Post-processing**	None	None (γ-corr. insets in B,D)				Deconv.	Deconv., GB 1	None		Deconv., GB 1		Deconv.		None	
**Illum. NA$_{eff}$**	0.83					0.83	1.1	0.83		1.1		0.83		0.83	
**AO**	On	Off		On		On				On	Off	On	Off	On	Off
**Registration**	No - 9 frames (1 Repetition)						No	No - 9 frames (1 Rep)				No		No - 9 frames (1 Rep)	
**SIM frame rate**	27.9 Hz						-	27.9 Hz				-		27.9 Hz	

Parameters	Fig. 3	Fig. S6A	Fig. S6B	Fig. S6C	Fig. S6D	Fig. 4A left	Fig. 4A right
**Sample**	Mouse: Thy1-GFP line M, *in vivo*					Mouse: C57Bl/6, *in vivo*	
**Sample prep.**	chronic cranial window					chronic cranial window, ChR2-GFP + CRE	
**Imaging depth**	25 µm	30 µm		25 µm	50 µm		
**Modality**	SIM					Widefield	SIM
**Illum. wavelength**	488 nm						
**Illum. power**	75 W/cm^2	54 W/cm^2		44 W/cm^2	125 W/cm^2	10 W/cm^2	
**Illum. time**	1 ms	200ms	1 ms	1 ms	2 ms	1 ms	
**Axial step**	0.2 µm	0.6 µm		0.2 µm	0.4 µm	0.2 µm	
**Axial projection**	A.I. over 1.0 µm	A.I. over 1.2 µm		A.I. over 1.4 µm	A.I. over 0.8 µm	A.I. over 1.0 µm	
**Post-processing**	None					Deconv.	None
**Illum. NA$_{eff}$**	0.83						
**AO**	On						
**Registration**	A,C: No; B,D: Yes	No - 9 frames (1 Repetition)		Yes - 27 frames (3 Repetitions)			
**SIM frame rate**	A,C: 27.9 Hz, B,D:9.3 Hz	27.9 Hz		9.3 Hz			

Parameters	Fig. 4C left	Fig. 4C right	Fig. 4E	Fig. S7	Fig. S8a	Fig. S8b
**Sample**	Mouse: Thy1-GFP line M, *in vivo*				Mouse: C57Bl/6, *in vivo*	
**Sample prep.**	Chronic cranial window		Chronic cranial window, KCl		Chronic cranial window, GCaMP6 + CRE + bicuculline	
**Imaging depth**						
**Modality**	Widefield	SIM	SIM	Widefield	Widefield	SIM
**Illum. wavelength**	488 nm					
**Illum. power**	44 W/cm^2		33 W/cm^2		125 W/cm^2	
**Illum. time**	1 ms				3 ms	
**Axial step**	0.4 µm				0 µm	
**Axial projection**	A.I. over 7.2 µm		A.I. over 1.6 µm		Single axial plane	
**Post-processing**	Deconvolution	None	None	Deconvolution	Deconvolution	None
**Illum. NA$_{eff}$**	0.83					
**AO**	On					
**Registration**	Yes - 27 frames (3 Repetitions)					
**SIM frame rate**	9.3 Hz					

Fig. S10. Summary tables of the experimental settings for all image data.

Movie S1. Dynamic structural imaging. Time-lapse movie with deconvolved widefield microscopy (dWF, left) and SIM (right) of a dendrite and its spines after KCl injection in the brain of a GFP-expressing mouse (Thy1-GFP line M). Scale bar: 2 μm.

Movie S2. Functional calcium imaging. Movie of GCaMP6 fluorescence variation with deconvolved widefield microscopy (dWF, left) and SIM (right) in a dendrite and its spine after bicuculline injection. Scale bar: 2 μm.

References

1. Wang K, et al. (2014) Rapid adaptive optical recovery of optimal resolution over large volumes. *Nat Methods* 11(6):625–628.
2. Wang K, et al. (2015) Direct wavefront sensing for high-resolution in vivo imaging in scattering tissue. *Nat Commun* 6:7276.
3. Turcotte R, Liang Y, Ji N (2017) Adaptive optical versus spherical aberration corrections for in vivo brain imaging. *Biomed Opt Express* 8(8):3891–3902.
4. Gustafsson MGL (2000) Surpassing the lateral resolution limit by a factor of two using structured illumination microscopy. *J Microsc* 198(2):82–87.
5. Gustafsson MGL, et al. (2008) Three-dimensional resolution doubling in wide-field fluorescence microscopy by structured illumination. *Biophys J* 94(12):4957–4970.
6. Kner P, Chhun BB, Griffis ER, Winoto L, Gustafsson MGL (2009) Super-resolution video microscopy of live cells by structured illumination. *Nat Methods* 6(5):339–342.
7. Wicker K (2013) Non-iterative determination of pattern phase in structured illumination microscopy using auto-correlations in Fourier space. *Opt Express* 21(21):24692–24701.
8. Lal A, Shan C, Xi P (2016) Structured illumination microscopy image reconstruction algorithm. *IEEE J Sel Top Quant* 22(4):6803414.
9. Wicker K, Mandula O, Best G, Fiolka R, Heintzmann R (2013) Phase optimisation for structured illumination microscopy. *Opt Express* 21(2):2032–2049.
10. O'Holleran K, Shaw M (2014) Optimized approaches for optical sectioning and resolution enhancement in 2D structured illumination microscopy. *Biomed Opt Express* 5(8):2580–2590.
11. Müller M, Mönkemöller V, Hennig S, Hübner W, Huser T (2016) Open-source image reconstruction of super-resolution structured illumination microscopy data in ImageJ. *Nat Commun* 7:10980.
